# The Chinese medicine Sini-San inhibits HBx-induced migration and invasiveness of human hepatocellular carcinoma cells

**DOI:** 10.1186/s12906-015-0870-6

**Published:** 2015-10-07

**Authors:** Hung-Jen Lin, Shung-Te Kao, Yu–Miao Siao, Chia-Chou Yeh

**Affiliations:** School of Post-baccalaureate Chinese Medicine, Tzu Chi University, Hualien, Taiwan; Department of Chinese Medicine, Buddhist Dalin Tzu Chi General Hospital, Min-Sheng Road, Dalin Town, Chia-Yi, 62247 Taiwan; School of Chinese Medicine, China Medical University, Taichung, Taiwan; Division of Chinese Medicine, China Medical University Hospital, Taichung, Taiwan; Department of Traditional Chinese Medicine Diagnosis, China Medical University Hospital, Taichung, Taiwan

**Keywords:** Sini-San, HBx, Matrix metalloproteinase 9, Tumor invasion, Nuclear factor-κB, Activator protein 1

## Abstract

**Background:**

Sini-San (SNS) is a formulation of four Traditional Chinese Drugs that exhibits beneficial therapeutic effects in liver injury and hepatitis. However, there are no reports describing its effects on the hepatitis B X-protein (HBx)-induced invasion and metastasis in hepatoma cells, and the detailed molecular mechanisms of its actions are still unclear.

**Methods:**

In this study, we investigated the mechanisms underlying SNS-mediated inhibition of HBx-induced cell invasion and the inhibition of secreted and cytosolic MMP-9 production, using gelatin zymography and Western blot analysis in a human hepatoma cell line (HepG2). Relative luciferase activity was assessed for MMP-9, NF-κB, or AP-1 reporter plasmid-transfected cells.

**Results:**

SNS suppressed MMP-9 transcription by inhibiting activator protein (AP)-1 and nuclear factor-κ B (NF-κB) activity. SNS suppressed HBx-induced AP-1 activity through inhibition of phosphorylation in the extracellular signal-related kinase (ERK) and c-Jun N-terminal kinase (JNK) signaling pathways. SNS also suppressed HBx-induced inhibition of NF-κB nuclear translocation through IκB and suppressed HBx-induced activation of ERK/phosphatidylinositol 3-kinase/Akt upstream of NF-κB and AP-1.

**Conclusions:**

SNS suppresses the invasiveness and metastatic potential of hepatocellular carcinoma cells by inhibiting multiple signal transduction pathways.

## Background

The hepatitis B virus (HBV) is associated with hepatitis and the development of liver cirrhosis, non-compensated liver disorder, and hepatocellular carcinoma (HCC) [1–3]. HBV genomic DNA comprises 4 partially-overlapping open-reading frames encoding core antigen, polymerase, and X protein [4]. Of these 4 HBV proteins, the X protein (HBx) has been implicated in the development of HCC [5–7]. HBx promotes tumor cell invasion and is involved in tumor metastasis and the recurrence of HCC [8–10]. Several studies have demonstrated that HBx affects the invasive ability of HCC through indirect activation of matrix metalloproteinases (MMPs) [8, 9, 11]. MMPs mediate extracellular matrix remodeling and are involved in a variety of pathophysiological processes, including tumor metastasis [12]. In HCC, an increased level of MMP-9 is associated with vascular invasion [13]. Several mechanisms have been proposed to explain the pro-oncogenic role of HBx, including activation of signal transduction cascades and transcription factors and interaction with a variety of cellular proteins [14–16]. The promoter region of MMP-9 has binding sites for the transcription factors AP-1 and NF-κB [17]. Stimulators, such as cytokines, control the expression of MMP-9 by modulating the activation of AP-1 and NF-κB through Ras/Raf/ERK, JNK, and PI-3 K-AKT/PKB signaling pathways [9, 14, 18, 19].

Many traditional medicines and phytochemicals have been considered as potential therapeutic candidates for controlling the invasiveness and metastasis of HCC [20–25]. Sini-San (SNS) comprises four prescriptions of Traditional Chinese Medicine (TCM) used in the treatment of various types of liver disease. This formulation was first described in “Treatise on Cold-induced Febrile Diseases (*Shanghan Lun*),” written by the famous Chinese physician Zhang Zhongjing (150 to 219 A.D. in the Chinese Eastern Han Dynasty). SNS has been used clinically routinely and has produced beneficial effects on liver injury [26, 27], hepatitis [28], chronic stress model [29], and Palmoplantar hidrosis [30]. The formulation shows both hepatoprotective activity and a virus clearing effect in patients with hepatitis [28]. Previous study showed SNS significantly inhibited tumor growth in HepG2 xenograft model [31]. However, the mechanisms for this effect are unclear. The aim of this study was to explore the mechanism underlying the inhibitory effect of SNS on HBx-induced cellular invasion and MMP-9 expression. In this study, we demonstrated that the inhibition of HBx-induced MMP-9 activity by SNS in hepatoma cells occurs via NF-κB and AP-1 signaling.

## Methods

### Extract of Sini-San (SNS)

Koda Pharmaceutics Ltd. (Taoyuan, Taiwan, ROC) provided the medicinal plants used to prepare SNS. Four raw herbs, Chaihu (*Radix Bupleuri Chinensis*), Baishao (*Radix Paeoniae Alba*), Zhishi (*Fructus Aurantii Immaturus*), and Gancao (*Radix Glycyrrhizae*) were employed at a ratio of 1:1:1:1. The procedure used to prepare the SNS decoction for this study is according to the method Koda Pharmaceutics Ltd. optimized. Briefly, raw materials from the four plants named above were combined to achieve a total weight of 100 g. Distilled water (100 ml) was added and the mixture was boiled for 2 h. After the volume was reconstituted to 100 ml with distilled water, the liquid was separated from the herbs and set aside. An additional 100 ml of distilled water was added to the herbs, and the 2 h boiling process was repeated. The volume was reconstituted to 100 ml, and the liquid was separated from the herbs and combined with the first water extract to achieve a final volume of 200 ml. The combined extracts were then filtered to remove particulate matter. This preparation (final concentration of 0.5 g/ml) was assigned a batch number of 32146903. For *in vitro* use, the herbal extract (SNS) was centrifuged at 7500 rpm for 30 min, and the supernatant was retained. A filtration through a 0.2-mm filter (Microgen, Laguna Hills, CA, USA) was performed to sterilize the preparation, which was then lyophilized and stored at −20 °C. The lyophilized product was reconstituted in methanol to a final concentration of 1 g/ml. To ensure the purity of SNS extract and to control the variation of quality for each batch, high performance liquid chromatography (HPLC) was performed.

### Reagents

For analysis of the signaling pathways involved in HBx-induced DNA-binding of AP-1 and NF-κB, we also treated HepG2-HBx cells with the p38 inhibitor SB203580 (SB), the MEK/ERK inhibitor PD98059 (PD), the JNK inhibitor JNKI, the IKK inhibitor BMS, and the Akt1/2 kinase inhibitor AKTI (Sigma-Aldrich) to block these pathways.

### Cell culture

The human hepatoma cell line HepG2 (Bioresource Collection and Research Centre, Taiwan) was maintained in Dulbecco modified Eagle medium (DMEM) (Life Technologies, Gaithersburg, MD, USA) and supplemented with 10 % fetal bovine serum (FBS) (HyClone, Logan, UT, USA). HepG2 cells were cultured in 25 cm^2^ flasks at 37 °C. The flasks were immediately capped and sealed with parafilm to minimize evaporation.

### MTT assay

Cell growth was measured using a modified MTT assay. Hepatoma cells were resuspended in medium (100 μL) in 96-well plates and cultured with or without DOX and SNS. After incubation for 24 h, MTT (20 μL) was added to each well and incubated further at 37 °C for 4 h. The supernatant was removed and DMSO (200 μL) was added to each well to solubilize the formazan product. The absorbance was measured at 470 nm using a microplate reader (Sigma). PT67, a retrovirus packaging cell line, was grown in Dulbecco’s modified Eagle’s medium supplemented with 10 % fetal calf serum, penicillin G (50 units/mL), streptomycin (50 μg/mL), and fungizone (1.25 μg/mL) at 37 °C in a 5 % CO_2_ incubator. HepG2 stable transfectants (HepG2-HBx) with doxycycline (DOX)-inducible expression of HBx-GFP were generated and cultured in complete MEM medium supplemented with 100 μg/mL G418 and 50 μg/mL hygromycin. The viability of variously treated HepG2-HBx cells was determined by MTT assay.

### Transfection and retrovirus infection

To investigate the relationship between HBx and MMP-9 expression, the vector pRT-HBxGFP was constructed and provided by Shin-Lian Doong [32]. DNAs were introduced into cells through transfection or retrovirus infection. Transfection was performed using the calcium phosphate DNA precipitation method according to the procedure described by Chen et al. [33]. The cells were transiently transfected with 5 μg of plasmid DNA of MMP9-Luc using SuperFect Transfection Reagent (Qiagen, Valencia, CA, USA). For retrovirus infection, media was collected from virus-producing PT67 cells and filtered through a 0.45-μm membrane. After addition of polybrene to a final concentration of 0.4 μg/mL, the whole mixture was poured onto the target cells. After incubation for 16 h, the virus-containing medium was aspirated. Cells were washed and incubated for 2 additional days before they were ready for selection or analysis.

### Wound-healing assay

HepG2-HBx cell lines were grown to 90 % confluence in 6-well plates at 37 °C in a 5 % CO_2_ incubator. A wound was created by scratching the cell monolayer with a sterile 200 μL pipette tip. The cells were then washed twice with PBS to remove floating cells, and serum-free medium was added. Photomicrographs of the wound were obtained at 100 × magnification.

### Invasion assay

Cell invasion was measured using Matrigel-coated film inserts (pore size, 8-μm) fitted into 24-well invasion chambers (Becton-Dickinson Bioscience, Franklin Lakes, NJ, USA). HepG2-HBx cells (5 × 10^4^ cells) were suspended in DMEM (200 μL) and added to the upper compartment of an invasion chamber in the presence or absence of DOX. DMEM (500 μL) was added to the lower chamber. The chambers were incubated at 37 °C in 5 % CO_2_. After 24 h, the filter inserts were removed, and the cells on the upper surfaces of the filters were removed with cotton swabs. Cells on the lower surfaces were stained with crystal violet, and the number of cells was determined by microscopy. Final values were calculated as the average of the total number of cells from 3 filters.

### Zymography

Gelatin zymography was used to determine the expression and activity of MMP-9 in HBx-treated human hepatoma cells in the presence or absence of SNS. HepG2-HBx cells were seeded onto 100 mm plates using serum-free medium and pretreated with DOX. SNS was added in different concentrations and incubated for 24 h. Conditioned medium was collected, and protein concentrations were determined using the Bio-Rad protein assay (Bio-Rad, Hercules, CA, USA). Culture supernatants were subjected to electrophoresis on gelatin substrate gels (10 % SDS-polyacrylamide gels containing 1 mg/mL of gelatin). The gels were treated with 2.5 % Triton X-100 for 30 min and incubated at 37 °C for 24 h in a buffer containing 100 mM Tris–HCl (pH 7.4), 0.15 M NaCl, and 15 mM CaCl_2_. The gels were stained with Coomassie Blue R-250 and then de-stained with water until clear zones appeared.

### Luciferase assay

Wild-type sequences were obtained for NF-κB (GGAATTCCCC) and AP-1 (TGAGTCA) DNA binding sites. Reporter plasmids (pNF-κB-Luc and pAP-1-Luc) were purchased from Stratagene (La Jolla, CA, USA). The MMP-9-Luc plasmid was kindly provided by Dr. C.K. Glass [34]. HepG2-HBx cell lines were co-transfected with reporter plasmids, treated with DOX for 20 h, and the luciferase activity determined. Briefly, HepG2-HBx cells were washed with PBS and lysed with 50 μL passive lysis buffer (Promega, Madison, WI, USA) at various time points after treatment. Lysates were transferred to 96-well plates, and the substrate was added (Promega, Madison, WI). A microplate reader (Synergy HT, Bio-Tek, Winooski, VT, USA) was used to assess the luciferase activity. Relative luciferase activity was calculated by dividing the relative luciferase units of MMP-9, NF-κB, or AP-1 reporter plasmid-transfected cells by the relative luciferase units of pGL3-Basic–transfected cells.

### Preparation of nuclear extracts and electrophoretic mobility shift assay (EMSA)

HepG2-HBx cells were treated with 100–800 μg/ml SNS. Nuclear extracts were prepared as described previously [35]. In brief, cells were stimulated, harvested by centrifugation, washed twice with cold PBS, and nuclear extracts prepared using the NE-PER reagent (Pierce, Rockford, IL, USA) according to the manufacturer's instructions. Biotin-labeled complementary oligonucleotides corresponding to NF-κB– and AP-1–binding sites were annealed. Biotinylated electrophoretic mobility shift assays (EMSAs) were performed as previously described [36], and gels were transferred to nylon membranes after electrophoresis. Membranes were blocked in blocking solution and proteins detected using alkaline phosphatase-conjugated streptavidin (Chemicon, Australia) followed by chemiluminescence assay (Roche, Germany).

### Western blot analysis

HepG2-HBx cells treated with or without DOX and 100–800 μg/ml SNS were lysed in 250 μL of sample buffer (62.5 mM Tris–HCl, 2 % SDS, 10 % glycerol, 50 mM dithiothreitol, and 0.1 % bromophenol blue; pH 6.8). We also collected the supernatants from cultures treated with SNS or 80 μM TPA. The supernatants were concentrated 40-fold using a Minicon filter (Millipore, Billerica, MA, USA) with a 15-kDa– cutoff pore diameter. Protein concentrations were determined using the BCA Protein Assay Kit (Pierce, Rockford, IL, USA). The cytoplasmic extracts (cytosol) were prepared using the Cytoplasmic Extraction Reagent (Pierce) according to the manufacturer's instructions. To assess the translocation of p65, the concentrations of p65 protein in cytoplasmic and nuclear extracts were detected by Western blot analysis. Proteins (10 μg for cell lysates and 40 μg for supernatants) were separated by 10 % SDS-polyacrylamide gel electrophoresis, and protein bands were transferred electrophoretically to nitrocellulose membranes. Membranes were probed with polyclonal antibodies against GFP, p65, MMP-9, EGFR, Akt, PI3K, IκB-α, phosphorylated IκB-α, JNK, phosphorylated JNK, p38, phosphorylated p38, ERK, phosphorylated ERK, and β-actin (Cell Signaling Technology, Beverly, MA, USA). Bound antibodies were detected using peroxidase-conjugated anti-rabbit antibodies followed by chemiluminescence assay (ECL System; Amersham, Buckinghamshire, UK) and autoradiography.

### Statistical analysis

One-way analysis of variance (ANOVA) was used to identify significant differences between the means (*p <* 0.05). If the means differed significantly, the Tukey-Kramer post-hoc test was used to compare the groups. Data are shown as the mean ± standard error of the mean (SEM).

## Results

### HBx expression in HepG2 cells and Cell Viability

**Fig. 1:**
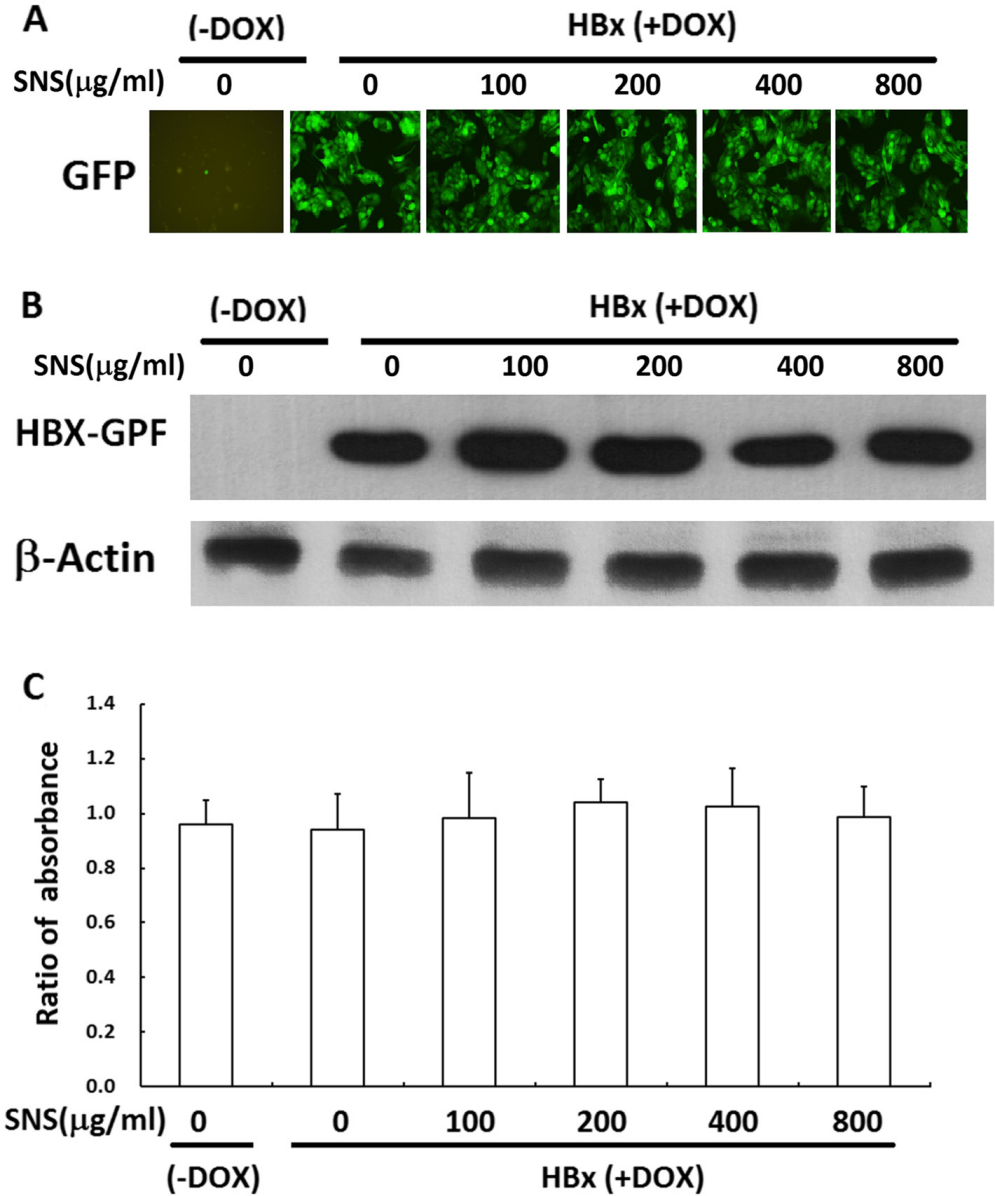
Establishment of constitutive HBx expression in HepG2 cells. HepG2 cells were transfected with pRT-HBx-GFP. After selection of G418-resistant colonies, cell proliferation with and without DOX treatment was assessed by MTT assay (**a**). HepG2-HBx-GFP cells with and without DOX were observed with a fluorescence microscope (**b**). Expression of the HBx-GFP fusion protein in HepG2-HBx-GFP cells with and without DOX treatment was determined by Western blot analysis using a GFP-specific antibody. β-actin was included as an internal control (**c**)

To determine whether HBx modulates the expression of MMP-9, we first confirmed whether HBx and the HBx-GFP fusion protein were expressed in HBx-transfected cells treated with or without DOX. GFP-fusion-protein expression was monitored using fluorescence microscopy, which detects fluorescence of the GFP protein (Fig. 1a). Furthermore, western blot analysis using an anti-GFP antibody showed HBx protein expression by transfectants (HepG2-HBx-GFP) only in the presence of DOX (Fig. 1b). In cells expressing GFP, fluorescence was observed in both the nucleus and cytosol. We therefore concluded that HBx-GFP was successfully transfected into HepG2 cells.

The cytotoxicity of HBx-GPF transfection and SNS treatment was evaluated using the MTT assay. No cytotoxic effects were observed for 100–800 μg/ml SNS without DOX, but cell numbers were slightly increased in those transfected with HBx-GPF in the presence of DOX (Fig. 1c).

### SNS inhibited HBx-induced invasion and migration in HepG2 cells

**Fig. 2:**
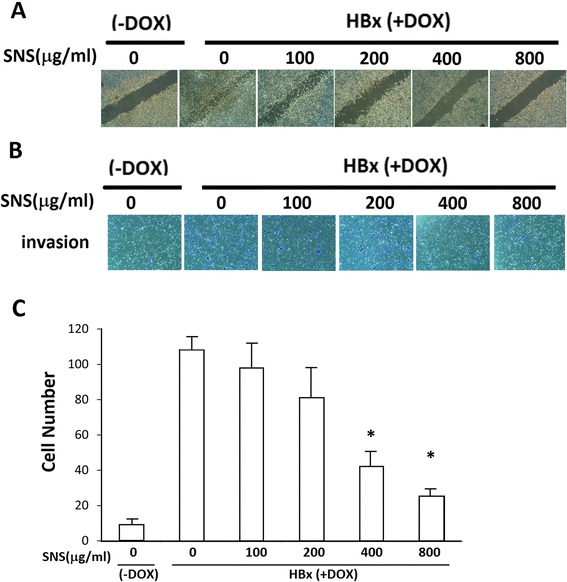
Effect of SNS on HBx-induced migration and invasion. **a** For the wound healing assay, HepG2-HBx-GFP cells with or without DOX were treated with different concentrations of SNS in 6-well plates and incubated for 24 h. Photomicrographs of the wounds were obtained under 100× magnification. **b** HepG2-HBx-GFP cells (5 × 10^4^) were resuspended in 200 μL of DMEM, added to the upper compartment of Matrigel invasion chambers, and treated with or without DOX and different concentrations of SNS. After 24 h, the cells on the lower surface of the insert were stained with crystal violet and counted under a microscope (200× magnification). **c** For cell quantification, HepG2 and HepG2-HBx-GFP cells were pre-treated with 100–800 μg/ml of SNS for 24 h. Results are expressed as fold-invasion and presented as the total number of invasive cells in treated cells relative to that in untreated cells. Values represent the mean ± SEM of 3 independent experiments. *p < 0.01 compared to HBx(DOX-treated) cells

*In vitro* invasion and migration assays were used to determine the inhibitory effect of SNS on the invasive potency of human hepatoma HepG2 cells. HBx transfection increased the invasiveness of HepG2 cells, which was inhibited by SNS in a concentration-dependent manner at 100, 200, 400 and 800 μg/ml (Fig. 2b, c). HBx-induced migration of HepG2 cells was inhibited in a concentration-dependent manner by treatment with SNS at 200, 400 and 800 μg/ml (Fig. 2a). Taken together, these data indicate that SNS inhibits HBx-induced cell motility *in vitro.*

### Effect of SNS on HBx-induced MMP-9 expression and activity

**Fig. 3:**
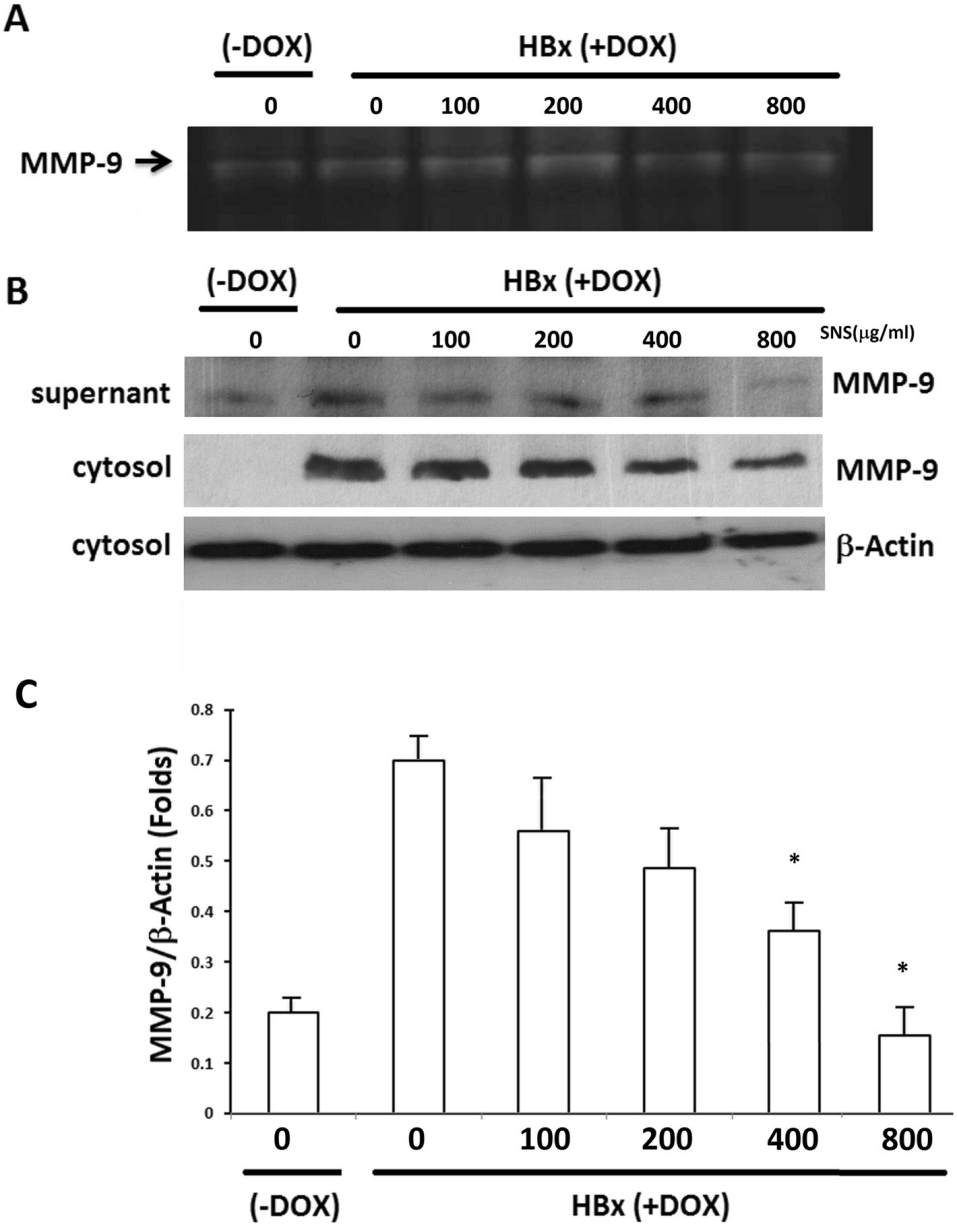
Effect of SNS on HBx-induced MMP-9 expression and activity. **a** Conditioned medium was collected from cultures after 24 h and analyzed by gelatin zymography. HepG2-HBx-GFP cells were cultured in 25 cm^2^ flasks, pretreated with 100–800 μg/ml of SNS for 1 h, then treated with DOX for 16 h. **b** Western blot analyses showed MMP-9 secretion into the culture medium (supernatant) and MMP-9. HepG2-HBx-GFP cells were pretreated with 100–800 μM SNS for 1 h, then treated with DOX for 16 h. Nuclear and cytosolic extracts were then prepared and subjected to western blot analysis using antibodies specific for MMP-9 expression. **c** Values of MMP-9 expression in the cytosol represent the mean ± SEM of 3 independent experiments. *p < 0.01 compared to HBx-expressing cells

Tumor invasion requires increased expression of MMP-9. To study whether the gelatinolytic activity of MMP in HepG2-HBx cells can be activated by HBx, we performed zymographic analyses. Figure 3a shows that treatment with DOX for 24 h significantly increased MMP-9 expression in HepG2 cells. HBx-induced MMP-9 expression was inhibited by 400 and 800 μg/ml SNS. This inhibition resulted from increased MMP-9 in the culture medium (supernatant) and cytosol (Fig. 3b, c).

### Effect of SNS on HBx-activated transcription of *MMP-9*, *NF-κB,* and *AP-1* promoters

**Fig. 4:**
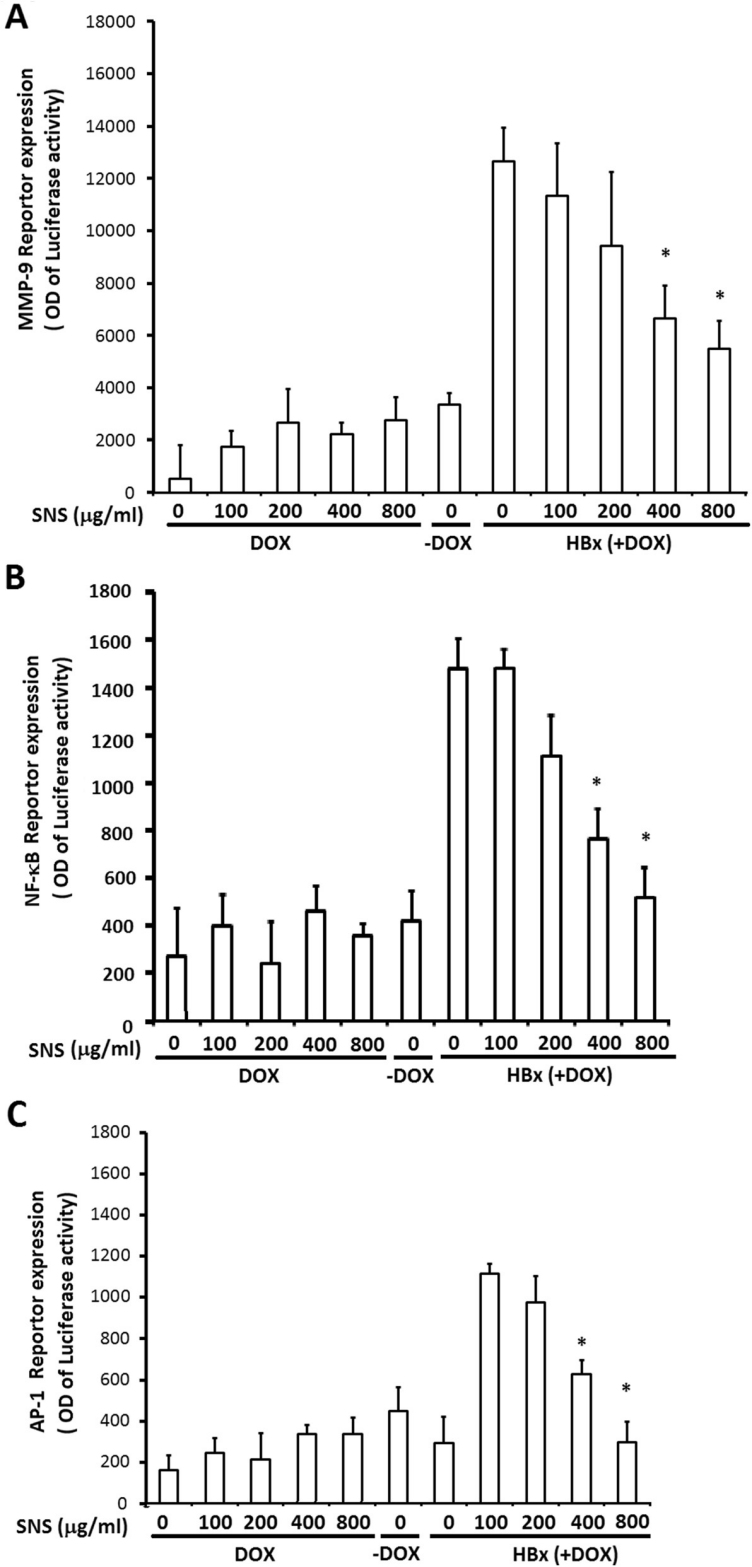
Effect of SNS on HBx -induced *MMP-9*-, *NF-κB*-, and *AP-1*-dependent luciferase reporter gene expression. HepG2-HBx-GFP cells treated with increasing concentrations of SNS were transfected with MMP-9- **a**, NF-κB- **b**, or AP-1 **c**-containing plasmids linked to the luciferase gene. HepG2-HBx-GFP cells were pretreated with 100–800 μM SNS for 1 h, then treated with DOX for 16 h. After 16 h treated with DOX, cell lysates were assayed for luciferase activity. Results are expressed as fold-activity over that of untreated transfected cells. Values represent the mean ± SEM of 3 independent experiments. *p < 0.01 compared to HBx (DOX-treated) cells

To determine whether the transcription of MMP-9, NF-κB, and AP-1 is regulated by HBx, we examined the promoter activity of these genes in HepG2-HBx cells co-transfected with p*MMP-9*, p*NF-κB,* or p*AP-1* by luciferase assay. The *MMP-9* promoter was activated by HBx at levels approximately 3.6-fold greater than that of control *MMP-9* promoter-transfected cells; this activation was suppressed by SNS in a dose-dependent manner and significantly suppressed by 400 and 800 μg/ml SNS (Fig. 4a). The *NF-κB* promoter was activated approximately 3.7-fold over that of *NF-κB*–transfected cells in response to HBx; this activation was suppressed by SNS in a concentration-dependent manner and significantly suppressed by 400 and 800 μg/ml SNS (Fig. 4b). Similarly, the *AP-1* promoter was activated approximately 4.1-fold over that of *AP-1*-transfected cells in response to HBx; this activity was also suppressed by SNS in a concentration-dependent manner and significantly suppressed by 400 and 800 μg/ml SNS (Fig. 4c).

**Fig. 5:**
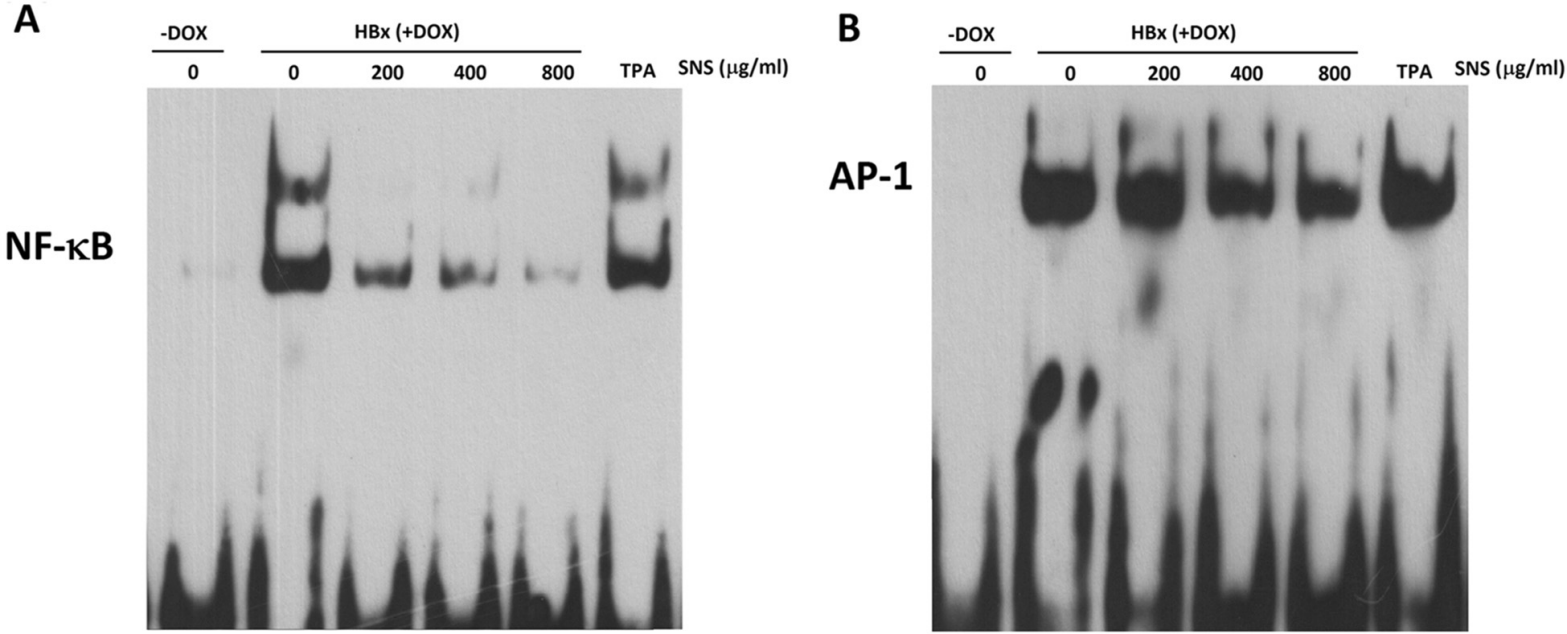
Effect of SNS on TPA-induced NF-κB and AP-1 activation in HepG2 cell. EMSAs were performed on nuclear extracts following HBx-induced NF-κB (**a**) and AP-1 (**b**) activation. HepG2-HBx-GFP cells were pretreated with 200–800 μg/ml SNS for 1 h, then treated with DOX for 45 min, followed by nuclear extract preparation

To determine whether the inhibitory effect of SNS in HBx-treated cells leads to NF-κB and AP-1 inhibition, the effects of SNS on HBx-stimulated NF-κB- and AP-1-specific DNA binding activity were examined. Using biotinylated EMSAs, HBx was shown to increase DNA binding of NF-κB and AP-1 after 45 min. Treatment with 400 and 800 μg/ml SNS inhibited HBx-induced DNA binding by NF-κB and AP-1 (Fig. 5a, b).

### Inhibitory effect of SNS on HBx-induced activation of MAPKs, IκB, and PI3K/ AKT

**Fig. 6:**
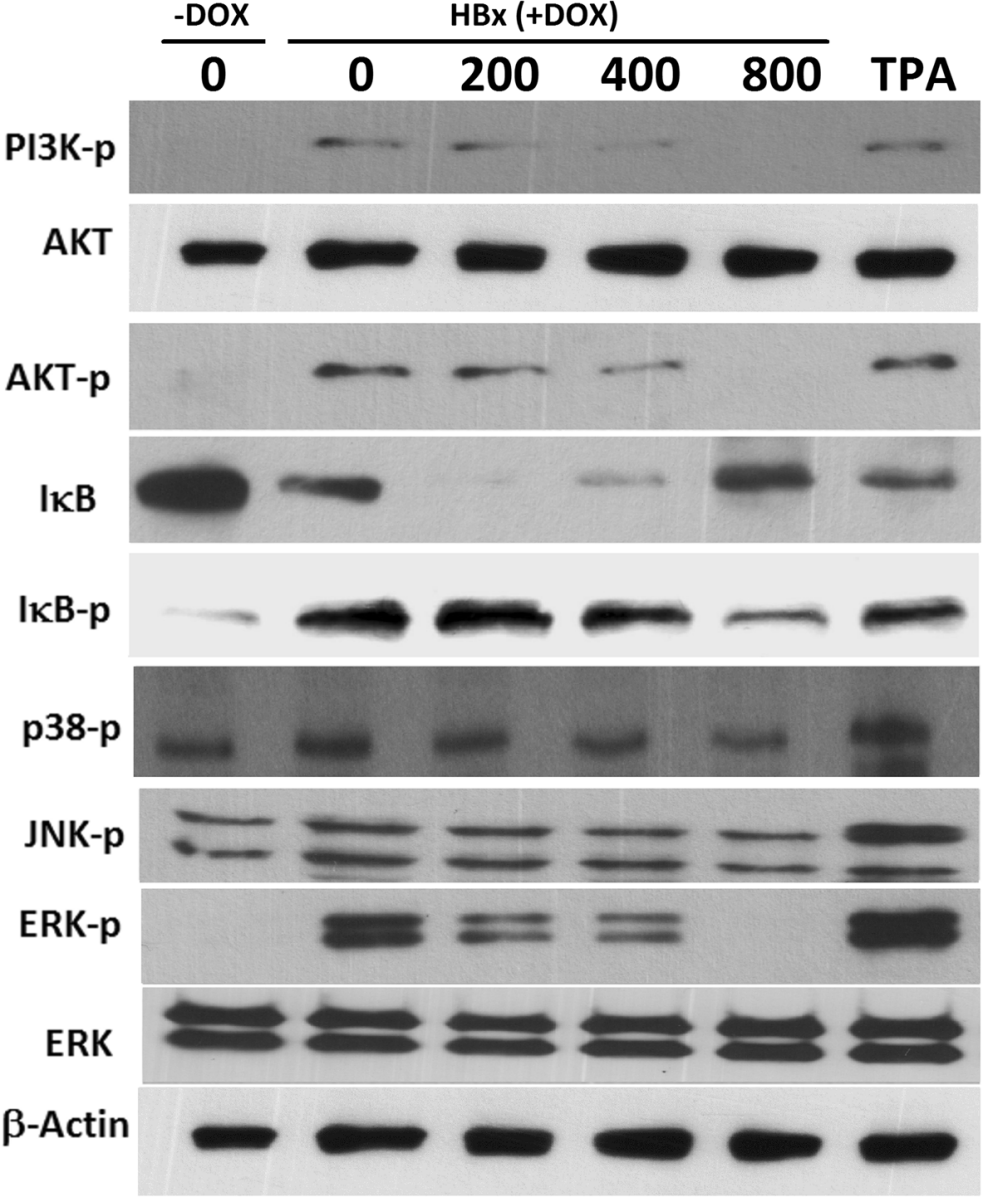
Effect of SNS on HBx-induced, MAPK, IκB, and AKT- signaling pathways. HepG2-HBx-GFP cells were pretreated with 200–800 μg/ml SNS before incubation with DOX for 45 min. Whole-cell lysates were then prepared and subjected to Western blot analysis using antibodies specific for phosphorylated PI3k, AKT, IκB, JNK, p38, and ERK in Western blot

MAPKs are known to regulate AP-1 and NF-κB activation via multiple mechanisms. Studies have shown that the MAPK, IκB, and PI3K/Akt signaling pathways are involved in HBx-mediated induction of MMP-9 [9, 37–39]. Therefore, we investigated the effects of SNS on HBx-induced phosphorylation of ERK, p38, JNK, IκB, and PI3K/Akt activity in HepG2 cells. Western blot analysis revealed that HBx expression alone caused a significant increase in the phosphorylation of ERK, p38, JNK, IκB, PI3K, and Akt compared to vehicle-treated controls; this phosphorylation was blocked by pre-treatment with SNS (Fig. 6). HBx-induced phosphorylation of MAPK, IκB, and PI3K/Akt was inhibited in cells treated with 400 and 800 μg/ml SNS.

## Discussion

Invasion and metastasis are fundamental properties of malignant cancer cells. Traditional Chinese herbal medicine could improve the prognosis and metastasis of cancer [40–43]. Metastasis of HCC is frequently observed, even at the early stages of the disease, and appears to be the main cause of shorten survival time of HCC patients [44]. The activation of signaling pathways by HBx may lead not only to the activation of transcription factors but also to profound cytological changes that contribute to tumor development, such as the acquisition of metastatic properties [8]. Elevated plasma levels of MMP-9 in patients with HCC have also been observed, especially in patients with macroscopic portal vein invasion [45]. HBV-negative HepG2, a highly invasive human hepatocellular carcinoma cell line , expresses moderate levels of MMP-9 [46]. HBx may induce MMP-9 expression and invasiveness in HBx-transfected cells [9, 11]. We hypothesize that SNS may suppress HBx-induced MMP-9 expression through activation of AP-1 and NF-κB. Thus, in this study, we investigated the effect of SNS on MMP-9 expression, cell invasiveness, metastasis in HBx-transfected cells, and the regulation of HBx–induced MMP-9 via signal pathways in HBx-transfected HepG2 cells.

Recent research has revealed the pharmacological actions of Chaihu (ingredients of SNS). Chaihu and its active components (saikosaponins) exhibited hepatoprotective [47], and anticancer effects [48, 49]. Baishao (*Paeoniae* radix) has been reported as a potential anticarcinogenesis agent [50] and anticancer effect [51]. Zhishi (*Fructus Aurantii Immaturus*) inhibited the translocation of NF-κB p65 [52]. Hesperidin (50 μM) and naringenin (25 μM), the major flavanones of Fructus aurantii [53], suppress TPA-induced tumor metastasis and *MMP-9* transcription by inhibiting NF-κB and AP-1 activity [23, 54–56]. Several studies have shown that MMP-9 expression is induced by HBx playing an important role in cancer invasion and metastasis in liver cells [9, 11, 37]. In the present study, we have shown for the first time that SNS suppresses HBx-induced cell invasiveness and MMP-9 expression, thereby decreasing HCC migration and invasion.

Stimulators increase the expression of MMP-9 through several signaling pathways, resulting in increased cell invasiveness. The promoter region of MMP-9 has both AP-1 and NF-κB binding sites. Stimulators, including cytokines, control the expression of MMP-9 by modulating the activation of transcription factors such as AP-1 and NF-κB through the Ras/Raf/ERK, JNK, and PI-3 K-AKT/PKB signaling pathways [9, 14, 18, 19]. The HBx protein has been shown to stimulate the activity of tPI-3 K-Akt/ PKB as well as ERK 1/2 in HBx-transfected cells [9]. Another report showed that HBx transfection induced NF-κB activation in HepG2 cells through inhibition of nuclear factor-κB kinase β (IKKβ) [57]. The present study showed that SNS effectively suppressed HBx-induced MMP-9 gene expression by suppressing the MAPK/AP-1 and PI3K/AKT/NF-κB cascades, with consequent suppression of tumor migration and invasion by human hepatoma HepG2 cells.

## Conclusion

We provide evidence that SNS exerts an anti-invasive and anti-migratory effect on HBx-activated hepatoma cells via the downregulation of PI3K/AKT, decreasing MAPK and IκB pathway signaling, NF-κB and AP-1 activity, and MMP-9 expression. SNS thus may have the therapeutic potential to inhibit the invasion and metastasis of HCC.
